# Biocomposites Based on Wheat Flour with Urea-Based Eutectic Plasticizer and Spent Coffee Grounds: Preparation, Physicochemical Characterization, and Study of Their Influence on Plant Growth

**DOI:** 10.3390/ma17051212

**Published:** 2024-03-06

**Authors:** Magdalena Zdanowicz, Marta Rokosa, Magdalena Pieczykolan, Adrian Krzysztof Antosik, Katarzyna Skórczewska

**Affiliations:** 1 Center of Bioimmobilisation and Innovative Packaging Materials, Faculty of Food Sciences and Fisheries, West Pomeranian University of Technology, Szczecin, Janickiego St. 35, 71-270 Szczecin, Poland; mpieczykolan@zut.edu.pl (M.P.); adrian.antosik@zut.edu.pl (A.K.A.); 2 Laboratory of Plant Physiology and Entomology, Department of Bioengineering, Faculty of Environmental Management and Agriculture, West Pomeranian University of Technology, Szczecin, Słowackiego St. 17, 70-953 Szczecin, Poland; marta.rokosa@zut.edu.pl; 3 Department of Chemical Organic Technology and Polymeric Materials, Faculty of Chemical Technology and Engineering, West Pomeranian University of Technology in Szczecin, Piastow Ave. 42, 71-065 Szczecin, Poland; 4 Faculty of Chemical Technology and Engineering, Bydgoszcz University of Science and Technology, Seminaryjna 3, 85-326 Bydgoszcz, Poland; katarzyna.skorczewska@pbs.edu.pl

**Keywords:** agricultural plastics, biocomposites, deep eutectic mixtures, fertilizers, spent coffee grounds, urea, wheat flour

## Abstract

In this study, we conducted the first plasticization of wheat flour (WF) with the addition of choline chloride:urea (1:5 molar ratio) eutectic mixture as a plasticizer and spent coffee grounds (cf) as a filler. Thermoplastic wheat flour (TPWF) films were obtained via twin-screw extrusion and then thermocompression. Their physicochemical characterization included mechanical tests, dynamic mechanical thermal analysis (DMTA), and sorption tests. XRD analysis revealed that the eutectic plasticizer led to a high degree of WF amorphization, which affected the physicochemical properties of TPWF. The results indicated that it was easy for the TPWF biocomposites to undergo thermocompression even with a high amount of the filler (20 pph per flour). The addition of the cf into TPWF led to an increase in tensile strength and a decrease in the swelling degree of the biocomposites. Biodegradation tests in soil revealed that the materials wholly degraded within 11 weeks. Moreover, a study of cultivated plants indicated that the biocomposites did not exhibit a toxic influence on the model rowing plant.

## 1. Introduction

Agricultural plastics (agroplastics) are materials that can be used in agriculture and horticulture for, e.g., mulching, as plant pots, seed tapes, or elements supporting plant growth [1]. Unfortunately, many of the commercially available products are made with oil-based plastics, e.g., mulch films, which are usually produced from polyethylene and seeding pots from polypropylene. Moreover, control release fertilizers are coated with synthetic resins. In particular, the latter can be a source of soil contamination with microplastics [2,3]. Small synthetic particles can be transferred to the soil by organisms or even incorporated into plants [3,4]. One of the solutions for this issue can be a replacement of oil-based polymers with compostable or biodegradable materials. Naturally abundant polymers, like polysaccharides and their derivatives, proteins, and polyhydroxyalcanoates, as well as some synthetically obtained polymers (e.g., polylactide—PLA, polycaprolactone—PCL or poly(butylene succinate—PBS), can be the source for the production of environmentally friendly agroplastics. It is worth highlighting that commercially available PLA is compostable only in controlled conditions, industrial composters, and water; its biodegradability is quite slow [5]. Starches or starchy flours have high potential for agricultural applications due to their natural origin, abundance, easy isolation from plants, and low cost. Starch and starch flours can be transformed into more thermoformable materials after their thermoplasticization via, e.g., extrusion with the presence of polar plasticizers [6,7]. During the process with high temperature and shearing forces, the structure of native starch that is originally semi-crystalline becomes more amorphous and strong -bonds of hydroxyl groups of polysaccharide chains are disrupted, forming new H-bonding between plasticizer molecules and the chains. These phenomena are common not only for starch but also for starchy flour, such as those widely used in food technology, like wheat flour (WF). WF, beyond its starch content, contains proteins (mostly gluten) in the range of ca. 10–24%, depending, e.g., on geographical region or level of nitrogen in soils [8], some lipids (ca. 1.1–1.4%) and fibers. The most common plasticizers for WF are polyols like glycerol and sorbitol [9,10,11,12,13], or their derivatives [14], but the mixtures with urea (U) can also be used [15]. The latter plasticizing systems have an advantage over glycerol due to urea utilization, which is known as a fertilizer. Moreover, U forms deep eutectic solvents—DES with other compounds (like ammonium salts [16,17,18], polyols [19,20], or sugars [20,21]). DES are mixtures with much lower temperatures of phase transition than their components [16] and can act not only as plasticizers but also as solvents [17,20,22,23], facilitating polymer processing [24]. Thus, materials plasticized with U addition are suitable for the production of agroplastics and a new generation of fertilizers. Thermoplastic starch-based materials are sensitive to moisture and exhibit quite weak mechanical properties, and one of the methods to improve their parameters is filler introduction and production of composites [25,26]. The most common fillers are cellulosic fillers [27] and clays [28]. However, food side products such as nut husks or shells [29,30], hulls [31,32,33], brewery byproducts [34], fruit pomace [35], or spent coffee grounds [36,37,38] can be applied. The advantage of using these additives is the fact that they are rich in many compounds, like polyphenols, organic acids, and minerals, which can add some extra functionality to biocomposites (e.g., increasing hydrophobicity and antioxidative properties).

In many households, spent coffee grounds (cf) are added to soil to support fertilization and soil amendment, and the reason for this is scientifically confirmed [39]. According to one study [40], 1 ton of green coffee beans generates 650 kg of cf, and ca. 2 kg of wet cf are obtained from 1 kg of soluble coffee during preparation. Coffee grounds waste is a source of many valuable organic compounds like fatty and phenolic acids, proteins, cellulose, hemicellulose, and lignin [41]. Protein content in cf is in the range of 12–17% and is much higher than that in roasted coffee (ca. 3%) [42]. The total content of nitrogen compounds like proteins, amino acids, and alkaloids (caffeine, N-methylnorharmane [43]) in coffee ranges from 8.5 to 13.6% and is at a relatively stable level between species or during roasting processes [41]. Coffee as a food byproduct can be applied for biomass gasification, biodiesel production, and sorbent-removing metal ions [41,44] as a filler for bioplastics [34,37,38,45]. However, there are only a few works related to thermoplastic starch (TPS) with cf or other coffee byproducts [46,47,48,49]. Additionally, few works described TPWF composites, i.e., with flax [50], sisal [12] cotton [10], or bran fibers [51], but there are no studies with thermoplastic wheat flour with cf.

This study aims to present biocomposites of natural origin with cf for fertilizing applications. Wheat flour was chosen as the biopolymer matrix because, in comparison with pure starch, it contains some proteins that can be an extra source of nitrogen. Moreover, this work, for the first time in the literature, presents the thermoplasticization of WF with an eutectic mixture rich in urea. Physicochemical properties of thermoplastic wheat flour (TPWF) and its biocomposites with cf, including mechanical tests, dynamic mechanical thermal analysis (DMTA), X-ray diffractometry (XRD), investigation of behavior in water and moisture, and biodegradation tests were studied, and compared. Additionally, due to their potential application as fertilizing materials, their influence on the physiological state of growing plants (model plant: the yellow dwarf bean—*Phaseolus vulgaris* L.), including chlorophyll “a” fluorescence and gas exchange parameters, content of proline, and photosynthetic pigment, were investigated.

## 2. Materials and Methods

### 2.1. Materials

Commercially available wheat flour—WF (type 500, Poznańska flour, Lubella Food, Lublin, Poland) with a moisture content of 9.5 wt% (nutrition value: carbohydrates 72%, proteins 11%, fiber 3.3%, fat 1.2%) was used. Eutectic plasticizer components were prepared according to work [52]: choline chloride—CC (p.a.) was supplied by Alfa Aesar (Kandel, Germany) and Urea—U (granules, ≥98%) by Chempur (Piekary Śląskie, Poland). Spent coffee grounds—cf (100% Arabica, finely ground coffee from Astra company, Nekla, Poland and Tschibo company, Marki, Poland) were collected by the employees of the CBIMO Unit. For the investigation of the physiological state of cultivated plants and the toxic influence of the biocomposites on the plants, a yellow dwarf bean “Złota Saxa” (*Phaseolus vulgaris* L.) with seeds purchased from the “Verve” company (Cracow, Poland) was used as the model plant. For the study on plants, “ATHENA” (Szczecinek, Poland) soil intended for the sowing and quilting was used.

### 2.2. Preparation of TPWF Films

Two methods of TPWF biocomposite composition were applied, depending on the filler introduction, according to our previous work [48], and prepared via two different methods depending on two ways of introducing the cf filler. The first method was as follows: plasticizer components: CC and U were mixed at a 1:5 molar ratio (60 parts per of the plasticizing mixture per 100 parts of the dry flour; pph), WF and dried cf (60 °C, overnight; 20 pph per dry flour) were mixed and kept in sealed LDPE bags for 20 h at RT before the extrusion. The final product from this composition is named TPWF/CCU/cf. For the second method, the reagents and components were the same, but another way of mixing was applied: cf was mixed with a hot eutectic mixture—DES (the same proportions, i.e., 20:60 per 100 parts of WF as in the first method) and then the whole mixture was kept at 80 °C for 120 min; then it was added into the flour and stored in sealed LDPE string bags for 20 h in ambient conditions before the extrusion. The sample obtained according to this method is named TPWF/CCU+cf. The premixture of DES and WF without cf was stored before extrusion in the same conditions as for the premixtures with cf (the final product is named TPWF/CCU).

After conditioning, all premixtures were processed with a twin-screw co-rotational extruder with L/D 40:1 and 10 heating zones (LTE20-40, LabTech, Phraeksa, Thailand). The temperature profile of the extrusion was set as follows: 60/100/105 × 9 °C, and the rotational speed was 85 rpm. Then, the extrudates were stored in LDPE bags for 1 week, granulated, and thermocompressed into sheets with dimensions 200 × 200 mm with the hydraulic press (Remi-Plast, Czerwonak, Poland) at 125 °C, with pressure of 153 bar for 10 s and cooled keeping the pressure until 80–85 °C was reached. The thickness of the films was 0.47–0.53 mm. Before further tests, the films were stored in a climate room at 23 °C, 50% RH, for several days. A scheme of the biocomposite preparation is shown in the previous work [49].

### 2.3. Tensile Tests

The mechanical properties of TPWF films were examined using a tensile machine (Zwick//Roell Z2.5, load cell 2.5 kN, ZwickRoell GmbH & Co. KG, Ulm, Germany) based on standard ASTM D822-02 [53]. The materials were cut into 10 mm wide strips. The tests were performed with an initial grip separation of 50 mm and a crosshead speed of 100 mm/min. Ten replicated samples for each film were measured, and the tensile parameters, i.e., Young’s modulus (YM), tensile strength (TS), and elongation at break (EB) parameters, were determined using the TestXpert II software.

### 2.4. DMTA—Dynamic Mechanical Thermal Analysis

The viscoelastic properties of the thermoplasticized materials were investigated using a Dynamic Mechanical Analyzer (DMA Q800, TA Instruments, New Castle, DE, USA). The storage modulus (E′) and the tangent of the loss angle (tanδ) as the temperature function were determined. The analysis was performed with a tension mode (specimen width 10–10.2 mm, thickness 0.48–0.52 mm), with a strain of 5 μm in the temperature range of −80–140 °C with a heating rate of 3 °C/min and a frequency of 1 Hz of the strain.

### 2.5. XRD Analysis

The filler (cf), native WF, thermoplasticized WF, and biocomposites were characterized using an X-Ray URD 6 diffractometer from Rich Seifert & Co GmbH (Freiberg, Germany) by using monochromatic X-ray diffraction with a wavelength of λ = 1.5406 Å (CuKα) in the 2θ angle range from 7 to 40 with step 0.02.

### 2.6. TGA—Thermal Gravimetry Analysis

TGA was used for the investigation of the thermal stability of neat wheat flour, TPWF/DES, and the biocomposites with TGA Q500 apparatus (TA Instruments, New Castle, DE, USA). Tests of ca. 20–24 mg of the samples were carried out on platinum pans in an air atmosphere, under 25 mL/min airflow, in the temperature range of 30–900 °C and heating rate of 10 °C/min.

### 2.7. Behavior in Moisture and Water

To investigate the behavior of the obtained materials in moisture and water (sorption, swelling, and solubility degrees), samples were cut into three pieces (625 mm^2^) per test and dried for ca. 120 min (100 °C) to constant mass. The dried samples were then weighed and placed in a climate chamber (BINDER KBF 115, Tuttlingen, Germany) at relative humidity—RH 50 ± 2%, temperature 25 ± 2 °C for moisture degree determination or immersed in distilled water for 24 h for the determination of swelling and solubility degrees. Then, the samples were weighed, and the parameters were calculated as in our previous work [49].

### 2.8. Biodegradability in Soil

The compostability and biodegradability degree of TPWF films were determined under controlled composting conditions using the methodology presented in the standard PN-EN 14046 [54]. The method was modified, as due to a release of ammonia during the test, the measurement of CO_2_ failed. The tested films were cut into smaller fragments (approx. 225 mm^2^). Then, the pieces were mixed in a ratio of 6:1 with the compost and incubated at a temperature of 25 ± 2 °C. The vessel was refilled with ¾ of a mixture to allow aeration with handshaking. The humidity of the compost was 55% ± 2 °C and pH = 7.3. In the following part of the study, the weight loss of the materials was measured. 

### 2.9. Investigation of the Toxicity and Influence of the Selected Biocomposite on the Physiological State of Growing Plants

For the investigation of the toxicity and influence of the WF-based materials on the physiological state of growing plants, the biocomposite film TPWF/CCU/cf was selected. In the first week of June, the yellow dwarf bean seeds (3 pots per variant, 2 seeds per pot) were sown in special soil mixed in a ratio of 4:1 with perlite. The biocomposite film was cut into small fragments (100 mm^2^) and mixed together with the prepared substrate before bean sowing. During the test, the water potential was maintained at −10 kPa in control conditions (optimal soil moisture) and −30 kPa in conditions of water deficit. The need for irrigation of plants was determined based on the contact soil tensiometers placed in the pots of the variants at a depth of ca. 20 cm. The studied variants (control and with the biocomposite) were irrigated using a drip line. Gas exchange parameters, chlorophyll “a” fluorescence parameters, and the determination of photosynthetic pigments and proline content were measured according to the methodology described in our previous work [49].

### 2.10. Statistical Analysis

For the statistical analysis of the results, ORIGIN software (ver. 2021, OriginLab) was applied using a one-way ANOVA test in a completely randomized design. The post hoc Tukey test was used to determine the significance of the differences between the means, with a significance level of α = 0.05. The same one-letter markings were applied to indicate means that did not differ statistically from each other.

## 3. Results and Discussion

### 3.1. Tensile Test Results

**Figure 1:**
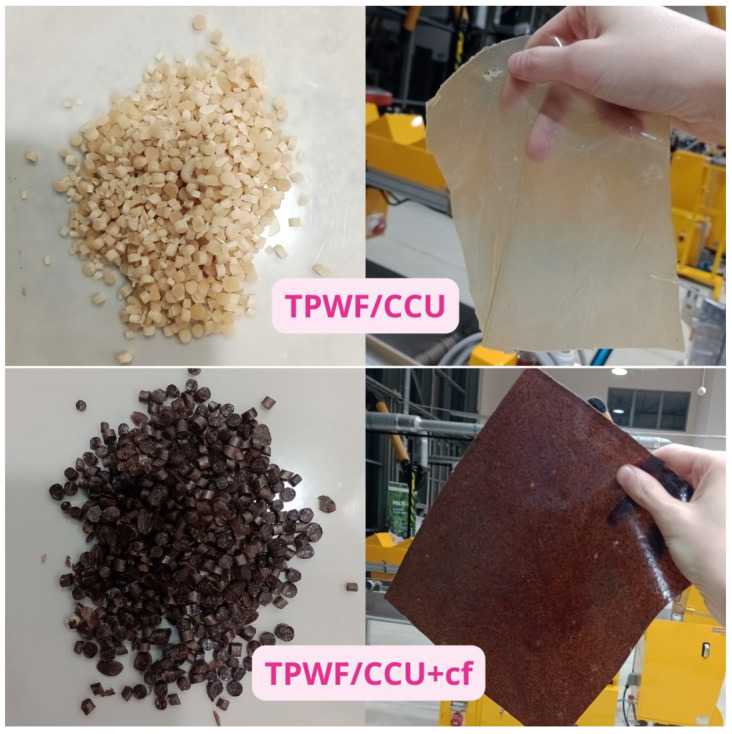
Extruded pellets (**left**) of TPWF/CCU and TPWF/CCU/cf (**right**).

The extrudate pellets were easily thermoformable via thermocompression molding to flexible films (slightly opaque TPWF/CCU and dark brown colored TPWF/CCU with cf). The appearance of the extrudate and the films are presented in Figure 1.

Results of the tensile investigation are shown in Table 1.

The films from thermoplasticized WF with eutectic plasticizer (CC:U 1:5 molar ratio) were uniform, and their flexibility was reflected in high EB (78%). This indicates that DES with high urea content was able to plasticize the wheat flour, effectively changing it into a more amorphous integral material. This study, for the first time, shows the application of DES for effective thermoplasticization of WF. In the literature so far, mostly thermoplastic starch, e.g., in works [17,18,20,23,24,49] or wheat gluten processed with DES presence [55,56,57] have been described. One exception could be Ma and coworkers’ study [15], where a mixture of urea and formamide was used to plasticize WF into materials with high EB, but the DES formation between co-plasticizers was not mentioned. We can only assume that there was some possibility of eutectic formation because the authors used a liquid mixture of the plasticizers, and according to the literature, it is known that these compounds mixed with other components can form DES [58]. Quite low TS (2.2 MPa) can be related to the high content of the plasticizer (60 pph) due to the higher content of plasticizers and the lower TS [59]. The addition of the spent coffee grounds, especially introduced as CCU+cf mixture into TPS/CCU affected the improvement of mechanical properties (higher YM and TS), leading to a decrease in EB, but still, the samples maintained some flexibility, despite high cf content (20 pph) indicating good dispersion of the filler in the thermoplasticized flour. Similar results were obtained in our previous work for corn starch [49]. It is worth highlighting that phenomenon because TPWF plasticized with glycerol with cotton fibers at 10% exhibited EB of ca. 14% [50] and TPWF with 20% of flex fibers at ca. 6% [10]. This low EB value indicates that the material was quite brittle. In another work [46] thermoplastic starch with cf exhibited a slight decrease in EB (down to 18% from 23% for TPS without the filler) for 20%. The increased YM (parameter related to the material stiffness) and TS for biocomposites are caused by the addition of organic solid filler that is compatible with the WF matrix and facilitated stress transfer between the filler and the matrix [50]. Better mechanical properties for TPWF/CCU+cf can be related to dissolved and extracted compounds from the solid coffee grounds into DES during heating of the DES+cf system that migrated into biopolymeric matrix acting as compatibilizers and co-plasticizer in the composite [49,60,61]. The differences in parameters between TPWF/CCU and the biocomposites are significantly different.

### 3.2. DMTA Results

**Figure 2:**
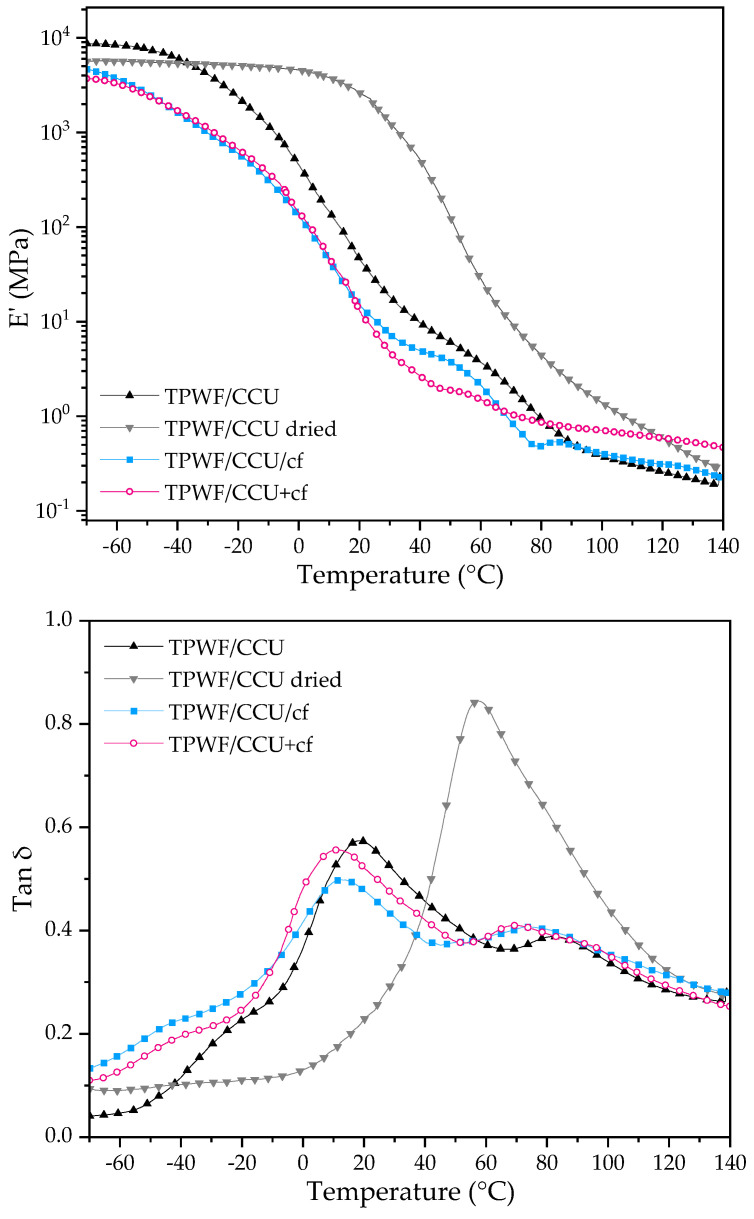
DMTA results for TPWF films. Storage modulus (E′) for TPS with CCU (**up**) and tan δ (**down**).

The DMTA results (the storage modulus—E′ and tan δ curves) are presented in Figure 2. To confirm an interpretation of the tan δ results, dried TPWF/CCU was added. For the conditioned films, E’ rapidly decreased with the temperature increase, and the values were low at elevated temperatures, which indicates their thermo-processable features [49,61,62,63]. Only for the dried sample was a pronounced drop of the parameter visible (at ca. 18 °C). This sample was quite brittle, indicating the key role of moisture or water (acting as the co-plasticizer) in the mechanical and viscoelastic properties of the biocomposites modified with hygroscopic DES. There are differences in TPWF/CCU and the composites. For the sample without the filler, E’ is lower than TPS with the filler at ca. 80 °C. Similar results were obtained in our previous work for TPS with DES betaine:urea 1:5 [49]. When comparing storage moduli at higher temperatures for composites, a difference between TPWF/CCU/cf and TPWF/CCU+cf can be noticed. As for TPS/DES/cf [49], it can indicate that CCU+cf premixtures form stronger bonds between DES and cf by partial extraction of compounds from the filler. 

Tan δ (dumping factor) is a measurement of how well a material can discard energy and is reported as the tangent of the phase angle. It informs how good a material will be at absorbing energy [64]. Three relaxation peaks can be observed on tan δ curves for conditioned samples. The first one at a temperature below −5 °C is a β-relaxation related to the motion of small molecules of external plasticizer and water. The second peak with high intensity is assigned to the α-relaxation of the plasticized biopolymeric chains [12], and the third peak with low intensity is an α’-relaxation peak assigned to moisture evaporation from the materials [63]. The peaks related to the movement of water molecules did not appear for the dried samples. Moreover, the α-peak for the dried sample is shifted toward a much higher temperature (ca 20 °C) than the conditioned analog (40 °C). Tan δ (dumping factor) is an effective method for the evaluation of interfacial bonding in composite materials, and the higher the tan δ peak, the higher the degree of molecular mobility [52]. The intensity of the α-relaxation peak is lower for composite TPWF/CCU/cf, and it can indicate some restriction of the polymer chain mobility from plasticized WF caused by the organic solid filler presence. On the other hand, a higher α peak of TPWF/CCU+cf in comparison with TPWF/CCU/cf can be caused by cf treatment with DES. U-based DES can be used for the extraction of different compounds from coffee byproducts [65,66]. During thermal pretreatment, some small molecules can migrate from the cf into DES, leading to an increase in the mobility of the polysaccharide [49,60]. The temperature of all the peaks is lower for composite films. It may be related to the filler treatment with DES during extrusion where high temperature and shear force are present and extraction of some molecules with lower molecular weight from spent coffee grounds into DES as well as solid particles presence that can disrupt the integrity of TPWF/CCU. 

### 3.3. XRD Analysis Results

**Figure 3:**
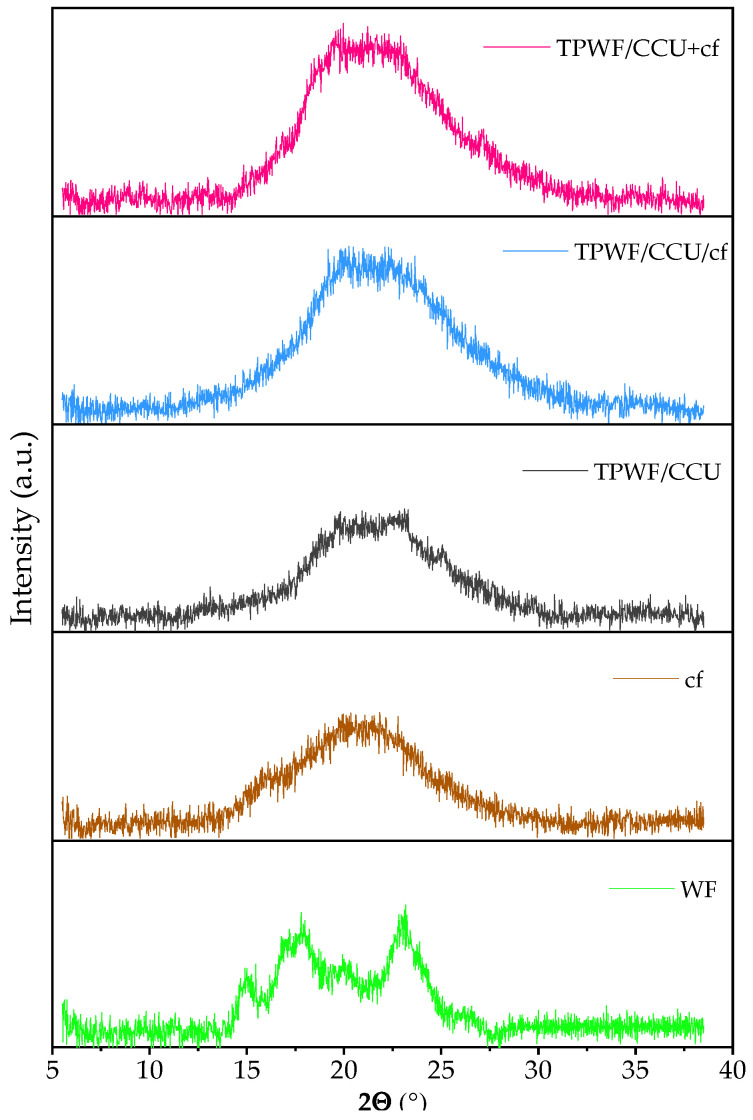
XRD pattern for native wheat flour (WF), spent coffee grounds (cf), thermoplasticized TPWF with CCU 1:5 and its biocomposites (TPWF/CCU/cf and TPWF/CCU+cf).

Figure 3 shows XRD patterns for native wheat flour, coffee filler, TPWF/CCU, and biocomposites. The diffraction peaks at 2Θ 14.9, 17.7, 20.2, and 22.6° reflect an A-type structure, which is characteristic for cereal starches [11]. Interestingly, both TPWF/CCU and its biocomposites have quite smooth, flattened patterns that indicate the highly amorphous structure of the materials. Only a barely visible peak at ca. 20.0° for biocomposites can be observed, with slightly higher intensity for TPWF/CCU+cf. It may be caused by the filler’s presence, which can act as an obstacle to full amorphization. On the one hand, in the case of CCU+cf introduced together into WF, DES mixed with cf is less available for the polysaccharide, while on the other hand, more dissolved fraction from the cf is released into the matrix and acted as an interfacial agent. Thus, the mechanical properties were better in the sample obtained via the second method (see results from mechanical tests and DMTA). A high degree of amorphization can confirm high values of EB (see mechanical test results) of TPWF films. This transformation of starch is characteristic of TPS plasticized with deep eutectic solvents [18,20] and mixtures with U [15]. On the contrary, TPWF plasticized with glycerol [9,11] or monoglyceride [14] still exhibited some remaining semi-crystal structure. TPWF plasticized with 20% glycerol exhibited a peak at 12.9° and a sharp peak with high intensity at 19.8° assigned to V_h_-type structure [9]. In the case of monoglyceride lipids, the higher the content of the additive, the lower the V_h_ peak intensity [14]. Comparing these results with those of the presented studies, U-based systems disrupt starch structure more effectively than conventional monoplasticizers. It can be caused by stronger H-bonding formation between U and starch as well as the dissolving activity of U-based eutectic mixtures [18,20,52].

### 3.4. TGA Results

**Figure 4:**
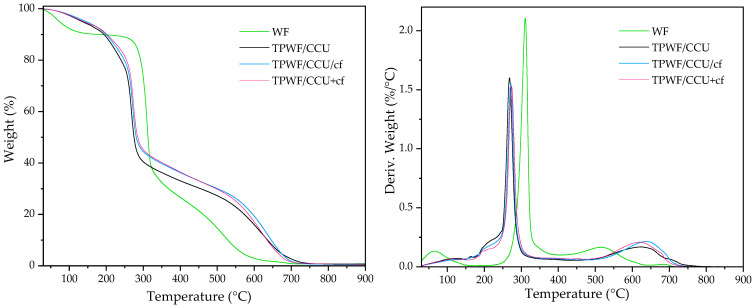
TGA results for native wheat flour (WF), thermoplasticized WF with DES (TPWF/CCU), and TPWF with spent coffee grounds (TPWF/CCU/cf and TPWF/CCU+cf).

Figure 4 shows the TGA curves of the materials based on wheat flour and its biocomposites. On the TGA curves, a three-step degradation process can be noticed, except for the unmodified powder of WF, where some extra sharp drop with weight loss of 9% at ca. 120 °C is observed, and it is attributed to moisture evaporation from the WF. It can also be seen that moisture in the thermoplasticized WF evaporated gradually because water molecules were bonded with the hygroscopic plasticizer [18]. However, this gradual loss is observed for higher plasticizer content [14]. The barely visible second step, with low intensity on DTG curves at ca. 198–209 °C, is assigned to the partial decomposition of the plasticizer [18,20]. The third step with the great drop in weight loss (DTG peak with high intensity for native starch 310 °C, TPWF/CCU 269 °C, TPWF/CCU/cf 272 °C and TPWF/CCU+cf 274 °C) is assigned to the decarbonization of the biopolymers. TPWF exhibited lower thermal stability compared to the native WF, which is caused by a more amorphous structure of the thermoplasticized WF [18,20,52] that is confirmed by XRD results and the presence of DES, which is rich in U [52]. The presence of the cf slightly affected the initial temperature of decomposition, and there is some difference in T_deg0_ for the two methods of the composite preparation. TPWF/DES+cf exhibited slightly higher thermal stability than TPS/DES/cf, but the differences are barely visible and insignificant. Similar results were obtained for analog materials prepared from corn starch [49], but in other works related to TPS or TPWF with organic plant fillers, a significant improvement of the materials was also not observed after filler addition [10,50].

### 3.5. Swelling, Dissolution and Moisture Sorption Degrees

Results from swelling, solubility (in distilled water), and moisture sorption degree at RH 50% are shown in Table 2. High swelling and dissolution degrees of TPWF/CCU (more than 300% and 47%, respectively) are related to the high degree of the amorphization of the biopolymer during its extrusion process in the presence of high plasticizer content [49] as well as high urea content in the eutectic system [49,52]. Compared to TPS analogs [49], the swelling degrees (256–305%) are slightly lower than TPS analogs (240–375%). It can be caused by the presence of gluten in WF, which is more hydrophobic than starch. The filler presence decreased swelling of the biocomposites without significant influence on dissolution degree.

### 3.6. Biodegradation in Soil

**Figure 5:**
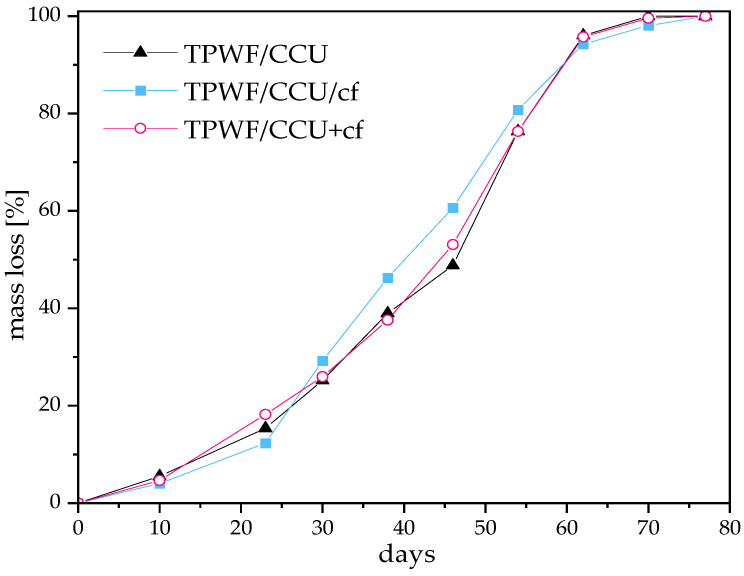
Mass loss curves for the biodegradation test in the soil of TPWF films and their biocomposites.

Figure 5 depicts biodegradation test results for the TPWF films buried in soil. In the first step of the test, samples swelled in the moisturized soil and started to defragment and vanish. As we can see, all the samples wholly degraded within 11 weeks, so this means that they degraded almost 2 weeks faster than the standard for compostable materials requires (90 days) [53]. There is no significant difference between the thermoplasticized WF and its composites. Comparing results with the TPS analogs [49], TPWF films took 1 week longer to degrade, and this may be caused by the lower swelling degree than in TPS films.

### 3.7. Influence of the Biocomposite on Toxicity and Physiological State of Growing Plant

The physiological state of the growing plant and the toxicity effect of the biocomposite were investigated on the yellow dwarf bean as a model plant. The results of the study are listed in Table 3, Table 4 and Table 5.

In Table 3, which presents the gas exchange parameters, it can be seen that the transpiration intensity for TPWF/CCU/cf decreased insignificantly in the studied plants except for a quite visible decrease in the substomal CO_2_ concentration. Comparing the gas exchange parameters, plants cultivated in the enriched substrate with biocomposite presence did not vary from plants grown in the control soil.

The F_V_/F_M_ parameter is considered the most reliable indicator of photosynthetic camera activity [67]. According to one study [68], the F_V_/F_M_ index in plants (Table 4) in their full development, under stress-free conditions, should exhibit values in the range of 0.78 to 0.84, and this value is dependent on genetic conditions [69]. In this work, studied plants growing with the presence of TPWF-based material in soil fit into this range, which suggests that this biocomposite did not exhibit a toxic effect on the functioning of the photosynthetic apparatus of the bean plants.

In the case of the content of assimilation pigments in bean leaves, only the content of carotenoids is significantly lower in plants growing on enriched substrate (Table 5). These pigments play an important role in the mechanisms that protect the photosynthetic apparatus from environmental stressors by dissipating excess energy or capturing reactive threnody species [70]. A decrease in their content may, therefore, indicate a decrease in the efficiency of the photosynthetic apparatus. On the other hand, the lack of significant differences between the control and biocomposite-supported growth in the case of proline content in the bean leaves suggests an absence of stress in plants [71] as TPWF/bran fibers our biocomposites are safe for cultivating plants [48].

## 4. Conclusions

The work presents the preparation and characterization of thermoplasticized wheat flour with eutectic mixture as the plasticizer and spent coffee grounds as the filler. Due to the plasticizing system being rich in urea, the final materials are designed to have a fertilizing function. This work presents the application of DES for WF thermoplasticization via extrusion for the first time. The results show that the CC:U mixture (1:5 molar ratio) effectively plasticized WF, leading to highly amorphous material, as mechanical tests, XRD, and DMTA results revealed. WF was transformed into thermoformable material that can be pressed into semi-transparent flexible films. Good thermoformability, even with high filler content (20 parts per 100 parts of WF), was confirmed by DMTA results (low E’ values at high temperatures). DMTA also indicated the role of water presence in the mechanical properties. The presence of cf led to increased tensile strength and decreased swelling degree of the biocomposites. All materials were made with natural-based components and fully degraded in soil over 11 weeks. The applied biocomposite had little effect on the physiological state of the bean plant (a slight decrease in the stomatal concentration of CO_2_ and the content of carotenoids in the leaves), and the toxic effect of the films was not demonstrated. The obtained results revealed that the biocomposite films could be used as eco-friendly agroplastics, or we could even call them agrobiocomposites with a fertilizing function.

## Figures and Tables

**Table 1:** Tensile test results.

Sample	Young’s Modulus (YM) [MPa]	Tensile Strength (TS) [MPa]	Elongation at Break (EB) [%]	Thickness [mm]
TPWF/CCU	38 ± 5.2 ^c^	2.2 ± 0.26 ^c^	78 ± 7.9 ^a^	0.51 ± 0.22
TPWF/CCU/cf	56 ± 9.2 ^b^	3.0 ± 0.34 ^b^	30 ± 4.7 ^b^	0.47 ± 0.03
TPWF/CCU+cf	91 ± 12.2 ^a^	4.2 ± 0.15 ^a^	34 ± 5.3 ^b^	0.50 ± 0.09

Homogeneous groups are marked with superscript letters.

**Table 2:** Swelling, dissolution, and moisture sorption degrees of the materials after 24 h of storage in distilled water or RH 50%.

Sample	Swelling Degree [%]	Dissolution Degree [%]	Moisture Sorption (RH 50%) [%]
TPWF/CCU	305 ± 10.2	47.4 ± 4.7	15.7 ± 0.07
TPWF/CCU/cf	286 ± 8.7	48.1 ± 1.8	15.3 ± 0.32
TPWF/CCU+cf	256 ± 6.3	45.5 ± 6.3	13.1 ± 0.31

**Table 3:** Gas exchange parameters of model plant leaves cultivated on soil substrate with TPWF/CCU/cf biocomposite presence.

Sample	Transpiration Intensity (mmol H_2_O·m^−2^·s^−1^)	Stomal Conductivity H_2_O (mol H_2_O·m^−2^·s^−1^)	Assimilation Intensity CO_2_ Net (P_n_)	Substomatal CO_2_ Concentration (μmol CO_2_·mol^−1^)
Control	0.393 ± 0.093 ^a^	0.036 ± 0.015 ^a^	2.508 ± 0.775 ^a^	414.67 ± 37.31 ^a^
TPWF/CCU/cf	0.385 ± 0.096 ^a^	0.032 ± 0.011 ^a^	1.917 ± 0.829 ^a^	314.42 ± 42.88 ^b^

Homogeneous groups are marked with superscript letters.

**Table 4:** Fluorescence parameters of chlorophyll “a” in model plant leaves growing on the substrate enriched with TPWF/CCU/cf biocomposite.

Sample	F_0_	F_M_	F_V_	F_V_/F_M_	TF_M_ (ms)	A_M_ (kbms)
Control	239.50 ± 18.75 ^a^	1119.08 ± 120.12 ^a^	895.25 ± 99.22 ^a^	0.782 ± 0.046 ^a^	866.67 ± 49.24 ^a^	52.89 ± 7.31 ^a^
TPWF/CCU/cf	223.17 ± 26.74 ^a^	1095.00 ± 121.48 ^a^	871.83 ± 131.10 ^a^	0.793 ± 0.041 ^a^	891.67 ± 25.87 ^a^	56.63 ± 9.73 ^a^

F_0_—Initial (zero) fluorescence; F_M_—Maximum fluorescence; F_V_—Variable fluorescence; TF_M_—Chlorophyll fluorescence growth time; A_M_—Area above the fluorescence induction curve. Homogeneous groups are marked with superscript letters.

**Table 5:** Content of assimilation pigments and proline in of model plant leaves growing on a substrate with TPWF/CCU/cf biocomposite presence.

Sample	Chlorophyll “a” (mg·g^−1^ FM)	Chlorophyll “b” (mg·g^−1^ FM)	Total Chlorophyll (mg·g^−1^ FM)	Carotenoids (mg·g^−1^ FM)	Proline (mg·g^−1^ FM)
Control	2.88 ± 0.19 ^a^	1.37 ± 0.20 ^a^	4.25 ± 0.22 ^a^	2.20 ± 0.02 ^a^	0.58 ± 0.05 ^a^
TPWF/CCU/cf	2.82 ± 0.18 ^a^	1.32 ± 0.09 ^a^	4.14 ± 0.22 ^a^	1.66 ± 0.15 ^b^	0.73 ± 0.19 ^a^

Homogeneous groups are marked with superscript letters.

## Data Availability

Data are contained within the article.
